# No compelling evidence that vaccination with streptococcus pyogenes group A carbohydrate elicits cross-reactive rheumatic fever autoantibodies

**DOI:** 10.1038/s41541-025-01272-0

**Published:** 2025-11-03

**Authors:** Tom Parks, Madeleine W. Cunningham, Allan Saul

**Affiliations:** 1 Department of Infectious Disease, Imperial College London, London, UK; 2 Department of Microbiology and Immunology, The University of Oklahoma Health Sciences Center, Oklahoma City, OK USA; 3 The Burnet Institute, Melbourne, Australia; 4 Department of Microbiology and Immunology, University of Melbourne, Melbourne, Australia

**Keywords:** Bacterial host response, Bacterial pathogenesis, Bacterial infection, Autoimmune diseases, Immunopathogenesis

## Abstract

We reviewed 70 years of research defining the specificity of anti-group A carbohydrate (GAC) monoclonal and polyclonal antibodies and antibodies raised against other *S. pyogenes* components reacting with GAC. While some rheumatic fever associated autoantibodies react with N-Acetyl-β-D-glucosamine sidechains of GAC and cross-react with tissues, these appear to be a consequence, not the cause, of autoimmunity. We propose GAC be considered further as a broadly protective group A streptococcal vaccine.

## Introduction

*Streptococcus pyogenes* or group A streptococci (GAS) infections are a growing concern due to increasing rates of invasive disease in several countries^1^, coupled with a substantial global burden of autoimmune sequelae^2^. Repeated infection with GAS can trigger acute rheumatic fever (ARF), especially among populations where GAS infections are endemic due to factors associated with socio-economic deprivation^3,4^. Major manifestations of ARF include rheumatic carditis and Sydenham’s chorea, both autoimmune conditions where antibodies and cellular immunity target human antigens in the heart and brain respectively^5^. However, even in hyper-endemic settings, only a proportion of the population appears susceptible to ARF, likely reflecting underlying host genetic susceptibility^6,7^. Despite association with a specific bacterial pathogen, the GAS antigen or antigens that trigger ARF remain unknown, complicating the development of a safe and effective vaccine. Moreover, because GAS exclusively infect humans, there are limitations to the animal models available to investigate the pathogenesis of rheumatic fever, including the role of GAC in the disease process^8^. Nonetheless, while appraisal of these models is beyond the scope of this review, they have been widely used in rheumatic fever research^9^, and a proportion of what is known about immune responses to GAS and its constituent antigens – including many of the issues discussed in this paper – have been based on studies in animals rather than infections in humans^10^.

**Fig. 1:**
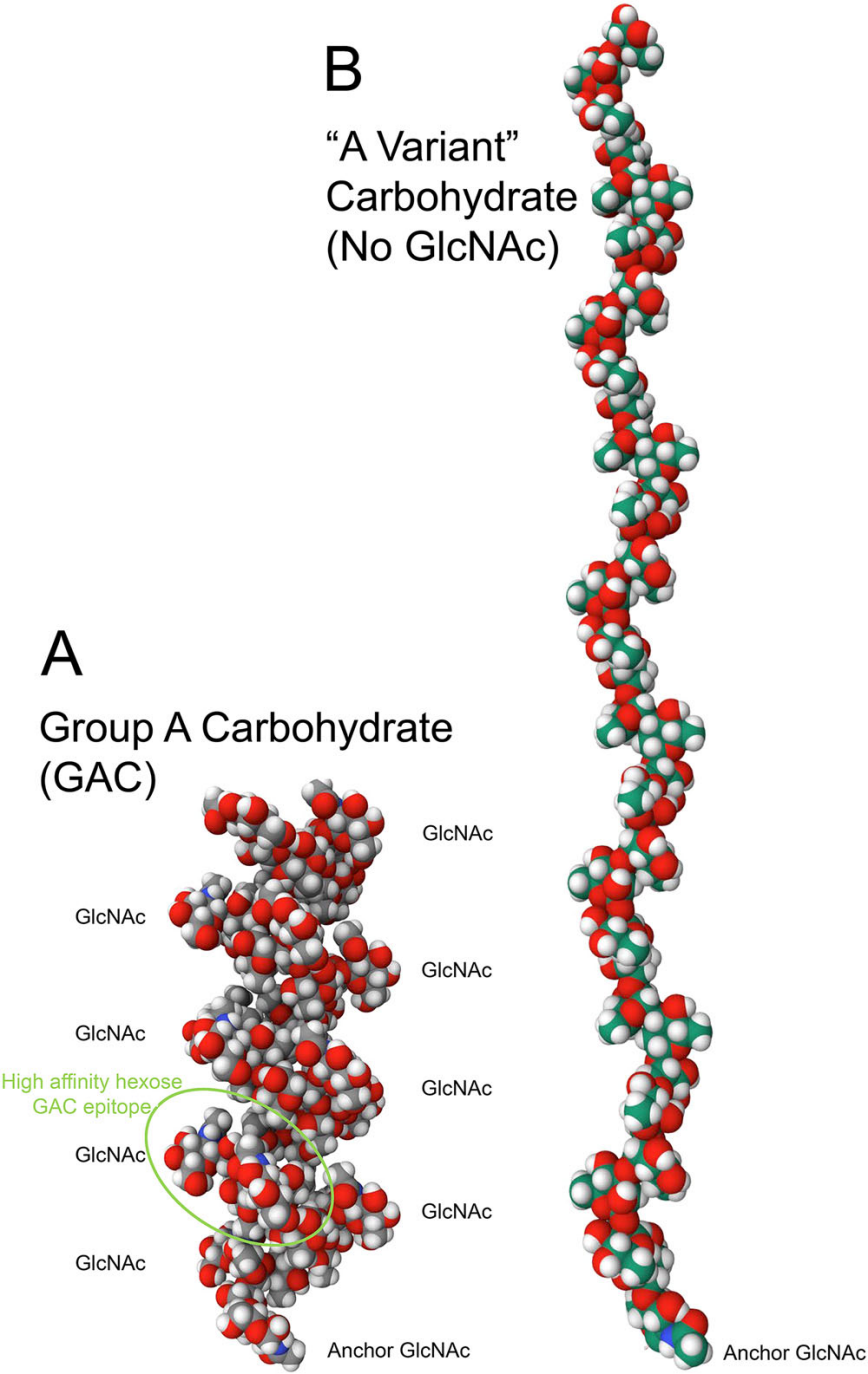
Space-filling model of carbohydrate structures. **A** GAC and (**B**) A Variant carbohydrate as modelled by Glycam^76^. Both structures have 18 repeats of the rhamnose disaccharide core - the average size of GAC isolated from GAS^15^. Both have the β(1, 4)linked GlcNAc that anchors the GAC to the peptidoglycan matrix^77^. The GAC structure is dominated by the GlcNAc linked to the core rhamnoses, Eight of the 18 GlcNAc in this structure are labelled. The green ellipse outlines one of the ~17 high affinity epitopes in this GAC identified using mAbs and other studies. It comprises 4 contiguous rhamnoses with 2 GlcNAc sidechains^35^. Not shown on the GAC are the glycerol phosphate attachments to some but not all GlcNAc sidechains^78^.

Streptococci are characterised by cell-wall anchored polysaccharides (Box 1), which in some cases can be used for serological classification^11^. Group A streptococci feature the Group A carbohydrate (GAC), a polymer of a 6-sugar repeat: a polyrhamnose backbone with N-acetyl glucosamine (GlcNAc) sidechains at alternating rhamnose (Fig. 1). Approximately a quarter of the GlcNAc side chains are decorated with glycerol phosphate^12^. Because it is the same on all GAS organisms, it makes it an ideal candidate for a comprehensive vaccine.

Data reported more than 50 years ago found higher levels and greater persistence of antibodies to GAC among ARF patients with carditis compared to matched controls^13^. More recently, this observation was replicated in a contemporary ARF endemic population, in which the authors demonstrated substantially elevated reactivity to a panel of GAS antigens including GAC also compared to matched controls^14^. The most likely explanation for these findings is that patients with ARF have accumulated reactivity to GAS antigens due to repeated infections, but the exaggerated response might also indicate an aberrant response to one or more of these antigens in the ARF patients. Nonetheless, extensive evidence from animal and in vitro studies with animal and human sera shows that GAC is an attractive vaccine candidate. Studies supporting its vaccine use have been recently reviewed^15^.

However, two areas of concern relating to GAC-based vaccines remain:

First, antibodies to the GlcNAc side chain of GAC may react with GlcNAc saccharides in human oligosaccharides^16^, generating autoantigens that react specifically to human GlcNAc-containing structures.

Second, antibodies to rhamnose and GlcNAc are found ubiquitously in relatively high concentrations in human sera^17^. These natural antibodies are constitutively expressed by germline B cells^18^. This class of antibody may be highly cross-reactive^19^, with each antibody binding to many targets, including GlcNAc. In mice, the number of germline B cells secreting anti-GlcNAc can be boosted by exposure to GAC and other antigens^20^. Therefore, boosting by GlcNAc in GAC may amplify these autoantibodies, potentially causing disease by binding to structures that do not contain GlcNAc.

Immunization of animals with GAS proteins (e.g., the intact M protein^21^ and an actin-like protein^22^) generates cross-reactive antibodies that recognize antigens in human tissues, especially cardiac myofibrils^23^. The molecular mimicry and auto-immunity associated with these antibody cross-reactions have been extensively reviewed^5^. Likewise, cross-reaction of T cell epitopes leading to T helper responses and infiltration of cytotoxic T cells is also well documented^24,25^. Although important, these protein-induced cross reactions are outside the scope of this review.

This narrative review (Box 2) examines the evidence surrounding risks of autoimmunity associated specifically with immunization with GAC. We start by

- reviewing the important study by Goldstein et al.^26,27^ that examined cross-reactivity between antibodies raised against bovine heart and GAC and vice versa;
- reviewing the immunology of the anti-GAC response including reactivity of anti-GAC antisera to human and animal tissues (i.e. by antibodies raised specifically against GAC);
- reviewing the properties of anti-GAS cross-reactive monoclonal antibodies (mAbs) from mice and humans, some of which react with GlcNAc (i.e. antibodies raised using GAS preparations most of which lacked significant GAC content).

We conclude with an alternate hypothesis that antibodies targeting GlcNAc (and thus GAC) reflect the emergence of autoimmunity detected by GlcNAc antigen presented as a hapten, rather than GlcNAc being the immunogen.

Box 1 Key GAS polysaccharides **Group A carbohydrate (GAC)**: GAC is composed of a polyrhamnose backbone with alternating α-L-(1 → 2) and α-L-(1 → 3) linkages and branching N-acetyl-β-D-glucosamine residues at alternate 3-positions^15^ (Fig. 1)

For the vaccine studies reviewed in this paper, one of two forms of GAC were used as the immunogen:

1. Most studies used “Streptococcal Group A vaccine”, a proteolytic digest of GAS cells usually with pepsin or occasionally with trypsin. This vaccine contained GAC covalently linked to peptidoglycan cell wall. Proteins such as the M protein that generate cross-reacting antibodies have been removed by digestion.
2. A conjugate of GAC with carrier proteins edestin or Typhoid toxin, or a synthetic polyelectrolyte. Prior to conjugation, GAC was prepared by cleaving the covalent link between GAS and the peptidoglycan cell wall with subsequent purification.

**Variant A carbohydrate:** In 1955 McCarty and Lancefield^30^ showed that repeated animal passages of GAS strains occasionally resulted in a new specificity – the “A variant” specificity – with an accompanying loss of GlcNAc. This variant A carbohydrate is a pure polyrhamnose polysaccharide.

**Peptidoglycan**: A polymer of alternating GlcNAc and N-acetylmuramic acid connected by a β-(1 → 4)-glycosidic bond and crosslinked with short peptides^30^. Immunization with GAS cell wall preparations (e.g. the “Streptococcal Group A vaccine” as used early papers) generated anti-peptidoglycan antibodies^36,80^ that must be considered in studies using GAS sera.

Box 2 Literature Search strategy Articles were identified for this Perspective by searching PubMed and Google Scholar for combinations of relevant terms including “*Streptococcus pyogenes*”, “Group A streptococcus”, “Group A carbohydrate”, “N-acetyl glucosamine”, “rheumatic fever”, “rheumatic heart disease” and “vaccination”. We also reviewed all papers listed by SCOPUS that cited three early key references: Kaplan and Svec 1964^82^; Goldstein et al.^26^; and Lyampert et al.^36^, We identified further articles from: personal collections of the authors, including the large number of references cited in substantive reviews by author MW Cunningham^10,83,84^; discussions with several experts in the field; and the bibliographies of articles that had been identified by other methods.

The literature reviewed for this Perspective article included publications up to March 2025. We did not confine the search to English and obtained English translation of several papers in Russian from the Gamaleya Research Institute in Moscow. We included relevant original research, in addition to narrative and systematic reviews, prioritising landmark publications in the field that have been widely cited. In several places, due to restrictions on the number of references permitted in the Perspective format, we have cited narrative reviews rather than original research that was not central to the issues discussed herein.

## Historical data: immunological cross-reactivity between heart and GAC – studies by Goldstein et al.^26,27^ and Kasp-Grochowska et al.^28^

In a widely quoted study that has been pivotal in defining the topic, Goldstein et al. in 1967, postulated that antibodies to glycoproteins from human heart valve cross-react with GAC^26^. Their study used rabbit antisera raised against bovine valve homogenates (BVH), bovine valvular structural glycoproteins (VSGP) and a chemical conjugate between GAC and a carrier protein (the hemp seed storage protein, edestin) to examine cross-reactions between heart antigens and GAC. Rabbits were vaccinated intramuscularly four times with VSGP or BVH emulsified with 0.5 mL Complete Freund’s Adjuvant (CFA). Anti-GAC sera were generated in rabbits by six intramuscular vaccinations with 15 µg/mL of GAC-edestin emulsified in 0.5 mL CFA followed by 3 iv injections of 20 µg of this conjugate. Notably, CFA contains 1 mg/mL of dried *Mycobacterium tuberculosis* cells and the use of large quantities of CFA for multiple vaccinations is unusual.

Antisera raised to BVH reacted by immunodiffusion with VSGP and GAC-edestin and gave bright immunofluorescence on GAS cells. Adsorption of the anti-BVH with VSGP prevented these reactions. Additionally, the results of cross-adsorption experiments with a single antiserum to GAC-edestin conjugate were reported. It gave a single immunodiffusion line when tested against VSGP or GAC-edestin and bright immunofluorescence on GAS. However, while pre-treatment of the anti-GAC antiserum with VSGP prevented the immunodiffusion reaction with VSGP, it did not prevent the immunodiffusion line with GAC-edestin nor the immunofluorescence on GAS.

A subsequent paper examined immunological relationships between GAC and a glycopeptide (“glycopeptide B”) released by partial pronase digestion of VSGP^27^. Glycopeptide B and GAC gave a line of identity by double diffusion, implying commonality between the antigens, but it is unclear in the paper which antisera was used.

Low concentrations of glucosamine and glucose gave partial inhibition of binding of anti-BVH antisera to glycopeptide B. Glucosamine, as well as the glycopeptide B, gave partial inhibition of binding of anti-GAC to GAC by immunodiffusion. This is unexpected for two reasons: first, the positively charged glucosamine is not a sugar found in GAC and, second, the concentration used (5 µM), was ~20 fold lower that the 100 µM K_D_ of the anti-GAC mAb with the highest affinity for GlcNAc in the series generated by Herbst et al. (see below)^29^. Inhibition by GlcNAc was not reported.

Working in an independent laboratory, Kasp-Grochowska et al.^28^ repeated these experiments. They extracted VSGP using both the original and a variation of the extraction procedure. The VSGP prepared with the alternative procedure (VSGP-D) had a much lower hydroxyproline (i.e. collagen) content than the original method. Results were reported using individual sera from eight rabbits vaccinated with BVH and eight vaccinated with killed GAS (but not with a GAC-conjugate or pepsin treated GAS).

Importantly, the authors could not replicate the results of Goldstein et al.: they were unable to demonstrate any specific cross-reaction between BVH antisera and GAC with serum from any of the eight rabbits. Unlike the Goldstein et al. sera, the pooled anti-BVH only gave weak immunofluorescence on fixed GAS and this could not be blocked with GAC.

They showed a weak immunodiffusion line between anti-GAS sera and VSGP in all rabbits tested, but this was present in both the pre-immune sera and the immune sera. (Goldstein et al. did not report experiments to test the pre-immune sera of their rabbits.) No immunodiffusion line was seen when VGSP-D was used. Kasp-Grochowska postulated that the weak immunodiffusion line with VSGP in the pre-bleed sera was due to the solubilized collagen in the preparation.

Kasp-Grochowska et al. used CFA only for the first vaccination and used incomplete Freund’s (IFA) adjuvant (lacking *M. tuberculosis* cells) for subsequent vaccinations. Moreover, rabbits vaccinated with just *M. tuberculosis* cells gave bright immunofluorescence on fixed GAS that could only be partially reversed by pre-adsorption with either GAC or A variant polysaccharide.

As summarized in Box 3, although the Goldstein et al. papers are frequently quoted as proof of cross-reactions between anti-GAC and heart tissues, the evidence is inconsistent, could not be replicated by Kasp-Grochowska et al., and there are credible alternative explanations, including that the cross-reactivity described in these papers was elicited by *M. tuberculosis*. These highly influential early studies have been superseded by more defined studies using mouse and human mAbs, which are discussed below.

Box 3 Summary of the Goldstein et al. and Kasp-Grochowska et al. papers

1. Evidence for cross-reactivity between VSGP and GAC was not reproducible:

- There were internal inconsistencies in the Goldstein et al. papers^26,27^ the inability of VSGP to compete for binding of anti-GAC to GAC by immunoprecipitation or by immunodiffusion, and the unexpectedly efficient competition of binding of anti-GAC to GAC by glucosamine, a sugar not present in GAC.
- Kasp-Grochowska et al.^28^, were unable to demonstrate any specific cross-reaction between anti-BVH antisera and GAC and vice versa. Despite the very strong reactivity of the anti-BVH sera on human myocardium, mitral valve, lymph node, kidney, and skin^85^, there was minimal cross-reaction by immunofluorescence between rabbit anti-BVH and GAS and this could not be blocked with GAC. A faint band was detected by double immunodiffusion between VSGP and GAC but this was present in the pre-immune sera. (Goldstein et al. did not report on testing their pre-bleed sera).

2. Since antibodies to mycobacterial antigens alone gave strong immunofluorescence on GAS^28^, the extensive use by Goldstein et al. of CFA for their vaccinations is a credible alternative explanation of the cross-reactivity described by them between their anti-BVH antibodies and GAS.

## Immunological specificity of animal polyclonal and monoclonal anti-GAC antibodies

In 1955 McCarty and Lancefield^30^ showed that vaccination of rabbits with heat killed, trypsin-treated GAC-variant lacking GlcNAc or wild type streptococci (i.e., containing GlcNAc) generated antisera that strongly reacted with the homologous polysaccharide but weakly or not at all with the heterologous polysaccharides^31^ (Box 1). These studies suggested that GlcNAc is an important part of the rabbit response to GAC and that GlcNAc shields the polyrhamnose backbone, preventing binding of anti-rhamnose antibodies (Fig. 1).

Briles and Davie (1975) showed that immunization of mice with pepsin digested GAS generated high levels of IgM antibody^32,33^. By using a haemolysis plaque assay, anti-GAC antibody secreted by single B cells recognized GAC sensitized red blood cells more efficiently than GlcNAc sensitized cells. The ratio of anti-GlcNAc vs anti-GAC plaques varied substantially from mouse to mouse (range from 4% to 96%, median 27%) suggesting that anti-GAC is more than just an anti-GlcNAc response. As judged by the isoelectric focusing pattern of anti-GAC antibodies from a single animal, the immune response was highly restricted^33^. They concluded that “*clonal commitment … does not result from competition among B cells for antigen*”, findings confirmed by subsequent studies with mAbs^34^ and sequencing of individual V genes from GAC binding mouse B cells^20^.

Herbst et al.^29^ prepared panels of mAbs from mice immunized with pepsin-treated, heat killed streptococci with GAC or A variant carbohydrate. A few anti-GAC mAbs also bound Group E and Group L polysaccharides. There was no cross-reaction between mAbs immunized with GAS and A variant GAS or vice versa, supporting the earlier rabbit polyclonal studies of McCarty and Lancefield^30^. As measured by fluorescence quenching, dissociation constants (K_D_) for soluble GAC binding ranged from <10 nM to 700 nM and for GlcNAc from 0.1 mM to 10 mM, consistent with the Briles and Davie^32^ results, showing that binding to GAC involved more than just the GlcNAc. Note that the K_D_ of the anti-GAC mAb with the highest affinity for GlcNAc (0.1 mM) is 20 times higher than the 5 µM concentration of glucosamine used in the Goldstein et al.^27^ study to inhibit binding of their anti-BVH antisera to GAC.

In recent data, reviewed by Pitirollo et al.^35^, multiple studies with polyclonal mouse and rabbit sera as well as two different anti-GAC mAbs demonstrated that GlcNAc alone was insufficient for high affinity binding. Affinity of binding to oligosaccharides increased with addition of rhamnose with the maximum affinity requiring at least a tri-saccharide and frequently the full hexasaccharide GAC repeat (Fig. 1). Results with human polyclonal anti-GAC sera were similar^35^. Saturation Transfer Difference-Nuclear magnetic resonance (NMR) spectroscopy indicated all four rhamnose molecules contributed to binding and that the acetyl groups were particularly important for high affinity binding^35^.

These studies show that GlcNAc is required, but not sufficient, for high affinity binding of defined antibodies to GAC. Importantly these studies reveal that assays based on GlcNAc-BSA, a commonly used surrogate for anti-GAC antibodies, should be interpreted with caution.

## Binding of polyclonal animal anti-GAC antisera to human and animal tissues

Several studies using polyclonal anti-GAC antibodies have been conducted to investigate their binding to human or animal tissues through immunolocalization, usually using immunofluorescence. Table 1 lists all the publications that we identified that examined binding of animal polyclonal anti-GAC antibodies on sections of human or animal tissues (Box 1).

**Table 1:** Immunolocalization studies with animal antisera raised by immunization with GAC

Study	Immunogen & vaccination	Target tissue	Localization results
Kaplan and Meyeserian, 1962^23^	Rabbits vaccinated with A typing reagent to produce anti-GAC (vaccination & adjuvant not specified) Rabbits vaccinated with washed streptococci and protein extracts of streptococci. Adjuvant: CFA followed by multiple injections without adjuvant	Immunofluorescence on smooth muscle cells from arterial walls.	No fluorescence with the A typing reagent. Conclusion: anti-GAC (A typing reagent) does not cross-react with tissue antigens Strong fluorescence on myofibrils with the anti-GAS sera completely abolished by adsorption with intact GAS cell walls and protein extracts of GAS but not by adsorption with high concentrations of GAC. Conclusion: Vaccination with whole cells and protein extract induced cross-reactive antibodies and the cross-reactive immunogen is protein and not GAC.
Lyampert et al., 1976^36^	Rabbits vaccinated with heat killed and pepsin treated GAS. No adjuvant. Sera extensively cross adsorbed with A variant GAS cells and sera then shown to only recognize GAC.	Immunofluorescence tested on a wide selection of human, guinea pig, rabbit, cattle tissues	*“No immunofluorescence on heart tissue or heart valves from a range of species”*. No surface immunofluorescence fluorescence on any tissue tested. Intracellular immunofluorescence in epithelial cells (see text for more detailed description) Conclusion: GAC presented as protease treated GAS cell walls induced strong anti-GAC antibody but no reactivity with heart or connective tissue
Ryzhikova et al., 1987^38^	Mice vaccinated with purified GAC coupled to a copolymer of acrylic acid and N-vinylpyrrolidone (GAC-PEL). No adjuvant.	Immunofluorescence on frozen sections of mouse skin, thymus, liver and sections of human thymus	*“Diffuse or perinuclear fluorescence of the cytoplasm of the epithelial cells. Strong reactions also were observed with cell nuclei on sections through epithelial tissues and liver tissue”*. Cytoplasmic immunofluorescence of epithelial cells could be prevented by pre-adsorption of the antisera with GAC. Nuclear immunofluorescence could not be prevented by pre-adsorption with GAC. Conclusion: Cytoplasmic immunofluorescence is GAC related. Nuclear immunofluorescence is not GAC related. Results concordant with rabbit polyclonal antibodies from the same group immunized with pepsin treated GAS^36^ and mouse mAbs generated against GAS-PEL^79^.
Sabharwal et al., 2006^80^	Rabbits vaccinated with GAC-Tetanus Toxoid conjugate. Adjuvant: CFA (first vaccination) then boosted with IFA	Immunofluorescence on human heart, brain, kidney, and liver tissue cryosections	*“We did not observe any binding of anti–GAS CHO antibodies to any of the tissues studied” (*it is clear from the paper that the “*anti-GAS CHO*” was GAC) Strong Immunofluorescence on human kidney & heart sections with a positive control: anti-proteoglycan antisera. Conclusion: GAC conjugate did not induce antibodies that cross-reactivity with heart, brain, kidney and liver.
van Sorge et al., 2014^81^	Rabbits vaccinated with wild type GAC-SP_0435 conjugate. No adjuvant	Immunohistochemical staining on human heart paraffin sections. ELISA on heart extract	No immunostaining of human heart sections. MAb anti-human cardiac myosin as a positive control gave strong staining. By ELISA, anti-GAC was negative on heart lysate. Control anti-GAS M1 protein was positive. Conclusion: GAC conjugate did not induce antibodies reacting with human heart

In the most comprehensive of these, Lyampert et al.^36^ tested sera from rabbits vaccinated with pepsin-treated GAS, adsorbing out non-GAC antibodies including anti-peptidoglycan, with A variant streptococci. The sera were tested by immunofluorescence on cryosections of: “*heart tissues of man, guinea-pig, rabbit, cattle and sections of human, bovine, and rabbit heart valves … the cornea and sclera of the rabbit and mouse eye … sections of thymus (nineteen specimens) and skin (eighteen specimens) from adult humans and human embryos (15–20 weeks of gestation). Thymus and skin tissues of rabbits, guinea-pigs, mice and rats were also studied*.”

The key finding as described by the authors was “*The absence of fluorescence of connective tissue elements, when the antibodies were applied on sections of heart tissues, heart valves, and cornea, indicates that cross-reactions between A-polysaccharide and connective tissue antigens cannot be detected*.” They found no surface immunofluorescence in any tissue examined although there was cytoplasmic fluorescence in basal epithelial cells of the skin, sclera and thymus.

These results are strikingly different to the earlier studies by Lyampert and colleagues^36,37^ based on vaccination of rabbits with killed and boosted with live GAS, grown through 10 passages in casein medium, which gave antibodies that strongly reacted by immunofluorescence and immunodiffusion with cardiac tissues and proteins. Similar to the adsorption studies of Kaplan and Meyeserian (1962)^23^, these earlier studies indicated that generation of cross-reactive antibodies by vaccination with GAS required the pepsin sensitive, i.e. protein, content of the GAS.

The Lyampert et al.^36^ findings based on pepsin-treated GAS are also consistent with the other studies with GAC-specific polyclonal antisera listed in Table 1, none of which found immunofluorescence on arterial smooth muscle cells, heart, brain or kidney tissues. In summary, no immunolocalization studies demonstrated reactivity of polyclonal anti-GAC antibodies with heart or brain tissues, nor any connective tissue or cell surface antigens. The only studies to show reaction of polyclonal anti-GAC antibodies with human tissue found binding limited to cytoplasmic antigens in basal epithelial cells from skin, sclera and thymus^36^, and one study, to nuclei^38^. Nonetheless, polyclonal sera derived from vaccinated animals are a crude tool with which to study a human autoimmune disease triggered by a human-specific pathogen.

## Mouse monoclonal antibodies prepared with GAC containing immunogens

These antibodies were prepared by immunizing mice with pepsin or trypsin digested, killed streptococci or with GAC conjugates with or without oil-based adjuvants (e.g. IFA).

Mouse monoclonals, termed the HGAC series, were produced by Nahm and colleagues^34,39,40^ using vaccination of A/J and B.C20 mice with killed pepsin-treated streptococci with selection for binding to GAC. The mAbs were almost exclusively IgM and IgG3, as expected for a T independent B-1 cell type response^19^. The heavy chain (V_H_) sequence of 16 of these has been published indicatingone of two V_H_ types (designated V_H_9 and V_H_39, after mAbs HGAC9 and HGAC39). Re-examination of these sequences using IgBLAST^41^ for this review, indicates all V_H_ sequences including both the “V_H_9” and “V_H_39” variants are from the IGVH6-3 locus or a closely related sequence.

Shikhman et al.^42,43^ demonstrated that HGAC49 (IgMκ) bound to keratin by ELISA and western blot and showed that two other mAbs, HGAC54 and HGAC78 (IgMκ) also bound to peptides from keratin, with the highest binding to keratin peptide “b1” that encodes a GlcNAc mimotope, also recognized by wheatgerm agglutinin. HGAC49 was tested for binding to a panel of cross-reactive antigens (myosin, actin, laminin etc.) used to characterize mAbs raised by immunizing mice with GAS membranes (see below). Other than keratin, the only other antigen recognized was the M protein of GAS serotype M6.

This study suggests the possibility of cross-reactivity between the GAS M protein and GAC. The hypothesis is supported by unpublished data from the Cunningham laboratory, which show that antibodies recognizing specific M protein peptides also react with GlcNAc when it is linked to bovine serum albumin (BSA). A relationship may exist between the alpha-helical M proteins and the GAC molecule, but cross-reactivity is observed only when GlcNAc is used as a hapten bound to a protein.

Turner et al.^44^ established that HGAC39 (IgG3κ) and HGAC78 bound to GlcNAc covalently linked to proteins via a serine or threonine (i.e. O-GlcNAc) but not to N-Glycans. Both mAbs gave the peri-nuclear and punctate cytoplasmic immunofluorescence on rat fibroblasts and hepatocytes expected from the distribution of O-GlcNAc. Similar fluorescence was observed by Shikhman et al.^42^ with HGAC49 on rat heart cells.

Rook et al.^45^ prepared a library of more than 200 mAbs that bound to GlcNAc-BSA from mice immunized with a trypsin-treated homogenate of killed streptococci. These mAbs were screened for binding to the glycoprotein, fetuin, and fetuin digested with neuraminidase and β-galactosidase to expose oligosaccharides with terminal GlcNAc. None of these mAbs bound to untreated fetuin indicating that these anti-GlcNAc mAbs could not react with normal human GlcNAc containing oligosaccharides. Only three of the more than 200 mAbs tested bound the neuraminidase-digested fetuin: i.e. almost all the anti-GlcNAc mAbs were unable to recognize terminal GlcNAc even in digested oligosaccharides.

Two of the mAbs, GN6 and GN7 that bound digested fetuin, were tested for immunofluorescence and immunoperoxidase staining to a range of normal and diseased human tissue^46^. No staining was observed on sections of non-diseased connective tissue, including synovial tissue from 12 joints, fascia from six areas, subcutaneous tissue from four areas, muscle (six samples) and brain (two samples). There was staining of epithelial cells from tonsils consistent with both cytoplasmic and surface locations. Cytoplasmic staining was also observed in salivary gland epithelial cells, skin keratinocytes and the Schwann cells in myelinated nerve trunks, which the authors speculated was due to O-GlcNAc. Granular macrophages from synovial fluid from patients with rheumatoid arthritis gave intense staining possibly due to processing of GlcNAc containing cellular debris.

Russian researchers^47–51^ prepared mAbs from mice vaccinated with pepsin- or trypsin-treated GAS. Cross-reactivity to mouse, bovine or human tissues depended on the fine specificity of the mAbs. At one extreme was mAb 4D/1 that bound to pepsin-treated Group A, A variant, C and L serotype streptococci^51^. By immunofluorescence, 4D/1 bound to cardiac connective tissue and all layers of human foetal skin. Immunofluorescence could be partially blocked by pre-treatment with purified A, C and L polysaccharides and fully blocked only with A variant polysaccharide, which was an unexpected specificity consistent with 4D/1 being an anti-rhamnose rather than anti-GAC antibody.

Other mAbs gave a similar pattern of fluorescence to that seen earlier by Lyampert et al.^36^ with polyclonal anti-GAC. Fluorescence was limited to cytoplasm of skin basal epithelia cells and certain other carcinoma-derived epithelial cells^49,52^. In a limited set of mAbs, Bazanova et al.^47^ reported mAbs that gave immunofluorescence on epithelial cells also bound to both Group A, A variant, and L polysaccharides^29^; mAbs that only bound to GAC and not to A variant or Group L did not bind to any tissues studied.

mAbs were also generated from mice vaccinated with GAC conjugated to a polyelectrolyte (GAC-PEL)^51^. Two of the mAbs (BI/2 and A5/2, both IgM) gave strong immunofluorescence with cell nuclei in mouse and human tissue sections. The immunofluorescence could be inhibited by double-stranded DNA, but not denatured DNA nor GAC and they did not bind to pepsin-treated GAS, i.e. these mAbs are not directed against GAC. Their specificity is consistent with earlier results obtained from vaccinating mice with the same GAC-polyelectrolyte construct where the polyclonal sera^38^ gave cytoplasmic and perinuclear fluorescence (consistent with reactions to O-GlcNAc^44^), as well as strong nuclear fluorescence (consistent with the anti-DNA specificity of these mAbs). The only anti-nuclear reactivity seen with any of the polyclonal or monoclonal anti-GAC antibodies came from animals immunized with this GAC-PEL construct. The authors speculated that the hybridomas expressing these IgM anti-DNA mAbs may “*have been obtained by polyclonal activation*”, triggered by the acrylic acid and N-vinylpyrrolidone copolymer “analogous to the action of LPS”. Anti-DNA and antinuclear reactivity of GAS antibodies were found only in mice^53^ and it is noteworthy that anti-nuclear antibodies are not a recognised feature of ARF.

More recent studies by New et al.^20^, sequenced individual B cells sorted by their specificity for binding fluorescent GAC with B cells from germ-free mice, from mice with a reconstituted microbiome or from mice vaccinated with pepsin-treated GAS. They found the B cells used a limited repertoire of V_H_ chains, predominantly IGHV6-3. They also showed that anti-GAC antibodies developed by active immunization with pepsin-treated GAS delayed development of type 1 diabetes. They proposed that anti-O-GlcNAc promoted more efficient clearing of pancreatic apoptotic cells without impacting survival in non-diabetic mice^54^.

In summary, some of the GAC-specific single cell antibodies or mAbs generated by vaccinating mice with protease treated GAS or GAC conjugates recognize O-GlcNAc. However, only a small proportion recognize GlcNAc in N-linked mammalian oligosaccharides and then only in partially digested or incompletely formed oligosaccharides. None have been shown to recognize normal N-linked oligosaccharides. Other than the GlcNAc mimicking peptide from keratin^43^, none have been shown to recognize non-glycosylated mammalian proteins.

## Mouse monoclonal antibodies prepared with immunogens containing limited GAC

**Fig. 2:**
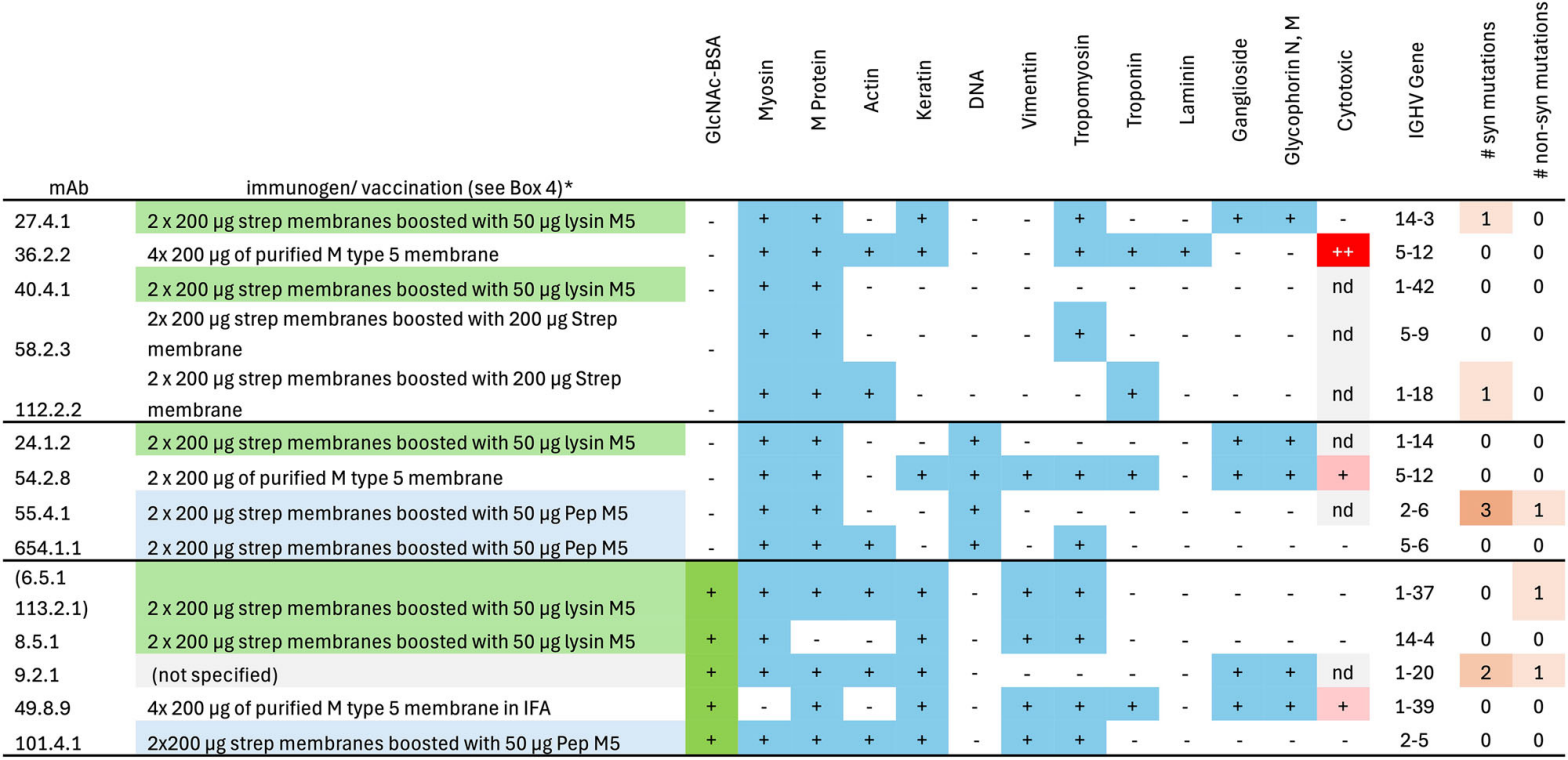
Summary of characteristics of Mouse anti-GAS mAbs. Vaccination strategies using antigens with no significant GAC or M protein have a white background; strategies using antigens containing GAC have a light green background; and strategies using antigens with no significant GAC but with M protein have a light blue background. Highlighting has also been used to emphasize mAbs that recognise GlcNAc-BSA (mid green background) and other antigens (mid blue background) as well as the level of cytotoxicity where tested (red background) and the number of mutations in the genes encoding the mAbs (brown). n.d. not determined.

These antibodies were prepared by immunizing mice with GAS membranes and boosted with either GAS membranes, lysin solubilized cell walls or a pepsin-digested M protein fragment with or without oil-based adjuvants (e.g. IFA) (see Box 4 and Fig. 2). They were generated by selecting hybridomas secreting mouse mAbs reacting with GAS by testing hybridoma supernatants by ELISA on plates coated with group A serotype M5 streptococci pelleted onto ELISA plates before fixing with glutaraldehyde^55^ and on ELISA plates coated with an extract of human heart^55^. Fourteen unique mouse mAbs were identified (Fig. 2)^56^ all of which were IgMκ. (Fifteen were identified, but two mAbs 6.5.1 and 113.2.1 have identical antibody gene sequences and specificities, were from the same mouse and are presumed identical.)

As shown in Fig. 2, these 14 mouse antistreptococcal mAbs bind a wide selection of human proteins. Three patterns of specificity were assigned based on binding to DNA and GlcNAc^56^. There was no significant association between three types of vaccination (Fig. 2) and binding to GlcNAc-BSA while binding to GAC was not reported. Five of the mouse mAbs bound GlcNAc-BSA conjugate and for the four of these tested, they required high concentrations (>100 mM) of soluble GlcNAc to inhibit this binding: a detailed titration curve for one (101.4.1) has been published with a 50% inhibition of binding at ~400 mM GlcNAc^42^. These concentrations are much higher than the 0.1 to 10 mM dissociation constant (K_D_) measured for the binding to GlcNAc of mAbs that bind GAC^29,57^, or the 0.7 mM and 30 mM, respectively, required to give 50% inhibition of binding of HGAC58 and HGAC78 to GlcNAc-BSA^42^.

The V_H_ and V_L_ nucleotide sequences of these 14 mAbs were determined^56^. As originally reported, these antibody V gene sequences had either germline or minimally mutated sequences. Reanalysis for this review, using IgBLAST^41^, further strengthened these original conclusions: now 11/14 hybridomas have BALB/c unmutated germline V_H_ and V_L_ sequences, and for the remaining three, only a single substitution was observed.

Three mAbs (36.2.2, 54.2.8 and 49.8.9) were cytotoxic in the presence of complement on cultured cells^58^ with 36.2.2 showing the strongest response and the only mouse mAb binding to laminin. In addition to their binding to human proteins, mAbs 36.2.2 and 54.2.6 neutralize polio virus, and 48.8.9 neutralized coxsackieviruses B3 and B4^58^. Of these three, only 49.8.9 bound GlcNAc-BSA and all three mAbs came from mice immunized only with GAS membranes (Box 4), i.e. a preparation that contained no significant quantities of GAC.

In conclusion, highly cross-reactive mAbs that recognize human, streptococcal, and viral proteins, can neutralize viruses in vitro and in vivo, and can be prepared from mice vaccinated with GAS membranes followed by boosting with GAS membranes, M protein fragment or solubilized cells. Some of these mAbs have cytotoxic activity against human or rat cells but it is unlikely that these mAbs resulted from vaccination with GAC.

Box 4 GAS preparations used for vaccinating mice or for in vitro stimulation of human lymphocytes to generate cross-reactive mAbs **GAS membranes**

Streptococci were fragmented in a mill with glass beads; the broken bacteria were centrifuged twice—first at low speed to pellet cell debris, including cell walls, and then at high speed to pellet the membranes^53,59,86^. Membranes used in these studies were not analysed but earlier studies indicated that these membranes contained no detectable rhamnose or hexosamine^87^, implying an absence of cell wall components or GAC. Other researchers found these membranes had significant T and B cell stimulatory activity on human peripheral mononuclear cells and tonsillar lymphocytes resulting in both cell division and a large increase in the number of antibody-secreting B cells^88^.

**Pepsin fragment of M protein**

GAS of serotype M5 were digested with pepsin, followed by pelleting of the cells and filtration of the supernatant, which was then concentrated^53,56^. This preparation contained various digested proteins but is unlikely to have contained significant GAC. It was emulsified in IFA for boosting mice.

**GAS whole cell digest**

GAS of serotype M5 were treated with mutanolysin, DNAse, RNAse and protease inhibitors^56,89^, which digested and solubilized the cell wall. The preparation was centrifuged, and the supernatant used. This preparation would have likely contained GAC. Presence of M protein was specifically demonstrated. These solubilized preparations were not evaluated for mitogenic activity, but other studies have shown that solubilized peptidoglycan-polysaccharide complexes purified from mutanolysin solubilized *S. pyogenes* cell walls have strong mitogenic and B cell activation activity for mouse B cells^90^

## Human mAbs prepared from healthy subjects as well as GAS, ARF and RHD patients

The fusions leading to these human mAbs falls into four groups (see also Box 4):

Set 1: The PB and T series mAbs derived from peripheral blood lymphocytes (PBL) from a cellulitis patient (PB series) or tonsillar lymphocytes from a patient with recurrent GAS pharyngitis (T2 mAbs) or from normal individuals with low ASO titres (T1, T6 and T7 series)^59^. Lymphocytes were stimulated in vitro with pokeweed mitogen or a fragment of the type 5 M protein released by pepsin digestion of whole GAS cells (Box 4). Binding to GlcNAc-BSA or GAC were not reported: all bound to human heart extract, rabbit skeletal myosin and GAS membranes. Individual mAbs bound to one or more of a panel of autoantigens (e.g. actin, calf thymus DNA), or GAS M protein.

Set 2: Hybridomas were generated from PBLs from an individual with chronic GAS carriage (9.B12 and 2.H11)^60^ or ARF/rheumatic heart disease (RHD) patients (1.C3, 1.C6, 1.C8, 1.H9, 3.B6, 4.F2, 5.G3, 5.G7)^61,62^. Lymphocytes were stimulated in vitro with a GAS whole cell digest likely to have contained GAC and peptidoglycan (Box 4), prepared by dissolving GAS cell walls with mutanolysin before affinity chromatography on Wheat germ agglutinin. Cells were then simulated with pokeweed mitogen and selected for binding to GlcNAc-BSA and for lack of binding to BSA. Not surprisingly all mAbs bound GlcNAc-BSA but also bound human skin keratin. Some also bound cytokeratin 8 and 18 (1.C8, 2.H11), human cardiac myosin (1.C8, 1.H9), rabbit skeletal myosin (4.F2, 5.G7), vimentin (1.C8), laminin (1.C3), and heat aggregated immunoglobulin (5.G7).

Set 3: Two closely related mAbs (10.2.3 and 10.2.5) were produced from tonsillar lymphocytes from a patient with recent GAS pharyngitis, stimulated in vitro with GAS membranes (Box 4) then pokeweed mitogen^63^ and selected for binding to GAS. These two mAbs have identical V_H_ chains, differing by one amino acid in the V_L_ chain, and have very similar binding profiles^61^.

Set 4: Three mAbs derived from PBL from a patient with Sydenham’s chorea (24.3.1, 31.1.1, 37.2.1). Lymphocytes were stimulated in vitro with streptococcal membranes, but not with pokeweed mitogen^64^ and selected for binding to GAS. All mAbs bound GlcNAc-BSA and lysoganglioside GM1 but had no detectible binding to double-stranded DNA, collagen, actin, human cardiac myosin, skeletal myosin and laminin. By immunohistochemistry, these mAbs bound to the surface of the human neuroblastoma SK-N-SH cell line.

Additionally, one of these mAbs (24.3.1) also bound tubulin^65^ and dopamine receptor D2^66^. Cross-inhibition of binding showed the antibody combining site was highly likely to be genuinely cross-reactive: lysoganglioside GM1 inhibited binding of 24.3.1 to tubulin. Functionally, 24.3.1 binds to cultured neuroblastoma cells^64^, and with the appropriate cell targets, activates CAM kinase II^64^ and elicits dopamine release^67^, which can be inhibited by GlcNAc-BSA. Targeting of dopaminergic neurons by this mAb and its mouse IgG equivalents in transgenic mice may provide a mechanistic insight into the neurological symptoms observed in Sydenham’s chorea^66,68^.

In summary, sets 1–3 but not Set 4 came from cells simulated in vitro with pokeweed mitogen. Sets 1, 3 and 4 were from cells that had not been stimulated in vitro with GAC or other GlcNAc containing antigens. However, all mAbs tested (i.e. Set 1 not tested), bound GlcNAc-BSA. This is not unexpected since the initial screening of most of the hybridomas included binding of their mAb to GlcNAc-BSA.

Three mAbs from Set 1 were IgG but all of the remainder were IgM. The V_L_ and V_H_ sequences of the some of the mAbs in Set 2^61^ and all the mAbs in Sets 3^61^ and 4^65^ have been reported, showing minimal changes from the closest germline sequences. With updated databases, reanalysis for this review shows the homology to germline sequences is even closer. For example, the V_H_ sequence of mAb 37.2.1 (accession number DQ779566) now has a 100% match with the germline sequence IGHV3-64 * 02 (V) and IGHJ2 * 01 or IGHJ3 * 01 or IGHJ3 * 02 (J).

All human mAbs were compared to the IgG specificity of the sera from the human patient and found to have similar reactivity as the IgG responses found in the sera of these patients from which the human mAbs were derived. Studies have been published on both the heart and the brain cross-reactive autoantibodies which demonstrate their IgG responses with human tissues and group A streptococcal antigens^64,66,69^.

## Implications for GAC containing vaccines

The mouse mAbs raised by vaccinating mice with GAS membranes and the human mAbs generated by in vitro stimulation of lymphocytes with GAS membranes and exposure to pokeweed mitogen provide a helpful model of the autoimmunity associated with ARF and other post-streptococcal diseases^10^. However, these monoclonal antibodies differ in multiple critical ways from the antibody responses generated by vaccinating with GAC containing preparations.

The “anti-GAC mAbs”:

1. Have been generated by immunization with GAC preparations, primarily protease treated GAS cells;
2. Bind GAC and, where tested, bind GlcNAc with lower affinity than GAC or GAC oligosaccharides, but still at substantially higher affinity than the “cross-reacting mAbs”;
3. Where sequenced, exclusively, use IGHV6-3 or closely related heavy chains, as also observed in most individually sequenced GAC binding antibodies from V genes from mouse B cells^54^;
4. Showed no binding to the surface of normal tissues. Some bind cytoplasmic components consistent with binding to O-GlcNAc, but there is no evidence of extensive binding to non-glycosylated proteins or to human N-linked oligosaccharides.

By contrast “cross-reacting, anti-GAS mAbs”:

1. Are all generated from mice immunized in vivo or human cells in vitro from ARF/RHD patients exposed to immunogens mostly lacking significant GAC content and/or other GAS antigens and potent B cell mitogens;
2. Gave highly cross-reactive binding to a range of human proteins by ELISA and by immunofluorescence to multiple human tissues;
3. Use a wide array of very low or unmutated germline V_H_ and V_L_ sequences with none of the mouse anti-GAS mAbs using IGHV6-3 or related V_H_ genes;
4. Have either a lower affinity for GlcNAc, or no detectible binding to GlcNAc;
5. In the case of the mouse mAbs, they show a degree of cross-reactivity and cytotoxicity that does not correlate with ability to bind GlcNAc (Fig. 2). The most cytotoxic mAb, 32.6.2 does not bind GlcNAc. Of the two mAbs with lower cytotoxicity, 54.2.8 and 49.8.9, one binds GlcNAc-BSA and the other does not.

The degree of cross-reactivity of the mAbs was apparently unrelated to vaccination with GAC containing immunogens: the median number of antigens in the cross-reactivity panel recognized by mAbs that did not bind GlcNAc was five compared to six for those that did bind GlcNAc (Fig. 2). This difference is not significant (*p* = 0.30, Mann-Whitney U test) implying that binding to GlcNAc-BSA is not required for cross-reactivity. Additionally binding to GlcNAc appeared related to binding to amino acid sequences which contained more amphipathic and aromatic amino acid residues potentially reflecting homology to alpha helical proteins^43^.

Additionally, the older data from rabbit and mouse anti-GAC (i.e. animals vaccinated with pepsin treated GAS or GAC conjugates) mirrored these findings: by immunofluorescence they showed no binding to surface antigens and no detectible binding to heart or brain sections.

## Conclusions

While concerns about autoimmunity from vaccination with GAS proteins may remain, a wealth of studies with both anti-GAC monoclonal and polyclonal antibodies do not support the hypothesis that antibodies to GAC play a causal role in ARF. Moreover, most of these studies were done by vaccinating with protease treated GAS cells while human vaccines in development use GAC conjugates (e.g. GAC conjugated to CRM_197_). Conjugates are expected to generate antibodies with higher avidity and a more restricted specificity than the T-independent B-1 type response generated by protease treated GAS cells^70^, further reducing the risk of cross-reactivity. The antibodies to GAC are important for their broad reactivity to all GAS and their protective ability. GlcNAc does not represent the intact GAC molecule as described herein, and GAC should be strongly considered an important potential vaccine for use in humans.

We propose an alternative explanation for the extensive cross-reactivity of anti-GAS mAbs: that these cross-reactive mAbs were generated by polyclonal activation of B cells exposed to GAS membrane fractions (mouse and human) containing undetectable GAC, combined with pokeweed mitogen in human mAbs. In this scenario, the high frequency of recognition of GlcNAc-BSA reflects the propensity at which germline antibodies recognize GlcNAc and the ready availability of GlcNAc-BSA as a reagent for assays^71^. Plausibly, a similar mechanism may contribute to ARF pathogenesis and may be partly reflected in several more recent observations regarding ARF pathogenesis including the role of germline antibody gene variation^72^, the striking elevation of the IgG3 antibodies^73^ and the heterogeneous nature of the autoantibody repertoire^74^.

Finally, rather than causing harm, immunization with a safe, effective GAC-containing vaccine that reduces the frequency and duration of exposure to GAS could reduce the risk and the devastating consequences of ARF/RHD and its sequelae. It is expected that the first human vaccine trials of a combination vaccine containing a GAC conjugate will start in the near future^75^, providing initial human safety and immunogenicity data as an important step in developing a broad based, safe and effective vaccine to protect children and adults from the devastating consequences of invasive GAS disease as well as ARF, RHD and other GAS autoimmune sequelae.

## Data Availability

No datasets were generated or analysed during the current study.
